# Germline Variants in Angiogenesis-Related Genes Contribute to Clinical Outcome in Head and Neck Squamous Cell Carcinoma

**DOI:** 10.3390/cancers14071844

**Published:** 2022-04-06

**Authors:** Dorota Butkiewicz, Agnieszka Gdowicz-Kłosok, Małgorzata Krześniak, Tomasz Rutkowski, Barbara Łasut-Szyszka, Krzysztof Składowski

**Affiliations:** 1 Center for Translational Research and Molecular Biology of Cancer, Maria Skłodowska-Curie National Research Institute of Oncology, Gliwice Branch, 44-102 Gliwice, Poland; agnieszka.gdowicz-klosok@io.gliwice.pl (A.G.-K.); malgorzata.krzesniak@io.gliwice.pl (M.K.); barbara.lasut@io.gliwice.pl (B.Ł.-S.); 2 I Radiation and Clinical Oncology Department, Maria Skłodowska-Curie National Research Institute of Oncology, Gliwice Branch, 44-102 Gliwice, Poland; tomasz.rutkowski@io.gliwice.pl (T.R.); krzysztof.skladowski@io.gliwice.pl (K.S.)

**Keywords:** head and neck cancer, polymorphism, FGF, PDGFR, MMP, TIMP, angiogenesis, treatment outcome, prognosis, radiotherapy, chemoradiotherapy

## Abstract

**Simple Summary:**

A high risk of relapse and treatment resistance are among the major challenges in locally advanced head and neck squamous cell carcinoma (HNSCC). Data show that common germline alterations in genes regulating angiogenesis may modulate treatment sensitivity, cancer progression, and prognosis, but relatively little is known about their role in HNSCC. Thus, our goal was to examine the effect of variation in these genes on survival outcomes in HNSCC patients receiving radiotherapy and cisplatin-based chemoradiotherapy. We identified genetic variants significantly affecting therapy results, constituting independent prognostic factors in these patients. Our results suggest that some polymorphisms in angiogenesis genes may be determinants of treatment efficacy and tumor aggressiveness in HNSCC, which may be of importance in standard therapy. These findings emphasize the potential value of the host genetic profile related to angiogenesis in assessing the risk of treatment failure.

**Abstract:**

Fibroblast growth factor (FGF)/FGF receptor (FGFR), and platelet-derived growth factor (PDGF)/PDGF receptor (PDGFR) systems, as well as some matrix metalloproteinases (MMPs) and their tissue inhibitors (TIMPs), are involved in various steps of angiogenesis. Data indicate that common germline variations in angiogenesis-regulating genes may modulate therapy results and cancer progression. However, whether these variants affect clinical outcome in head and neck squamous cell carcinoma (HNSCC) is unclear. Hence, we assessed the relationship between FGF/FGFR, PDGF/PDGFR, MMP, and TIMP genetic variants and treatment outcomes in HNSCC patients receiving radiotherapy (RT) alone or combined with cisplatin-based chemotherapy. In multivariate analysis, *FGF2* rs1048201 CC homozygotes showed a higher risk of death (*p* = 0.039), while *PDGFRA* rs2228230 T was strongly associated with an increased risk of locoregional relapse (HR 2.49, *p* = 0.001) in the combination treatment subgroup. In the RT alone subset, *MMP2* rs243865 TT carriers had a higher risk of locoregional recurrence (HR 2.92, *p* = 0.019), whereas *PDGFRB* rs246395 CC homozygotes were at increased risk of metastasis (HR 3.06, *p* = 0.041). The *MMP2* rs7201 C and *TIMP2* rs7501477 T were associated with a risk of locoregional failure in the entire cohort (*p* = 0.032 and 0.045, respectively). Furthermore, rs1048201, rs2228230, rs246395, rs243865, rs7201, and rs7201/rs7501477 were independent indicators of an unfavorable outcome. This study demonstrates that the *FGF2*, *PDGFRA*, *PDGFRB*, *MMP2,* and *TIMP2* variants may contribute to treatment failure and poor prognosis in HNSCC.

## 1. Introduction

Head and neck squamous cell carcinoma (HNSCC) is one of the most common malignant neoplasms in the world, very often diagnosed in an advanced stage [1]. Although progress has been made in the treatment of this cancer, locoregional and distant relapse occurring in a large number of patients is still a serious problem leading to poor survival outcomes. In HNSCC, decreased sensitivity, or resistance to treatment, is one of the major challenges in terms of patient prognosis. In locally advanced and unresectable HNSCC, radiotherapy (RT) and cisplatin-based chemotherapy (CT) are the mainstays of treatment [2]. Their effectiveness is highly influenced by hypoxia and dysregulated angiogenesis [3,4]. 

Angiogenesis is recognized as playing a crucial role in the development and progression of solid tumors [5]. Several growth factors are involved in different steps of new blood vessel formation. Angiogenesis is primarily mediated by the vascular endothelial growth factor (VEGF)/VEGF receptor (VEGFR) system; however, other proangiogenic growth factors, such as fibroblast growth factor 2 (FGF2, also known as basic FGF, or bFGF), are also very potent regulators of the process. FGF/FGF receptor (FGFR) signaling leads to proliferation, migration, and differentiation of endothelial cells and fibroblasts [6]. The FGF pathway may indirectly control angiogenesis by coordinating other growth factor signaling (e.g., VEGF) and various cell–cell interactions [7]. Platelet-derived growth factor (PDGF) is another angiogenesis-inducing cytokine that exerts its effects by interacting with PDGF receptors α (PDGFRA) and β (PDGFRB). The PDGF/PDGFR system is critical for the proliferation, migration, and recruitment of mesenchymal cells, including vascular smooth muscle cells, pericytes, and fibroblasts [8]. Both networks, FGF/FGFR and PDGF/PDGFR, are implicated in embryogenesis, tissue regeneration, and wound healing, and when deregulated, are also involved in tumor growth, survival, and metastasis [9,10]. High levels of these proteins have been associated with a worse prognosis in various cancers [6,8,11,12].

The structure and composition of the extracellular matrix (ECM) are important factors in the regulation of angiogenesis. Angiogenesis is accompanied by the degradation of the vascular basement membrane and ECM components by matrix metalloproteinases (MMPs). These calcium-dependent zinc endopeptidases, produced by stromal and tumor cells, are involved in inflammation, tumor invasion, and metastasis. MMPs are essential for tumor angiogenesis, as they participate in vascular remodeling, cell migration, and sprout formation [13]. Two gelatinases, MMP2 and MMP9, are believed to play particularly important roles in this process. They are known to activate and release proangiogenic growth factors (e.g., VEGF and FGF2) from ECM, as well as generate antiangiogenic molecules [14,15]. The enzymatic activity of MMPs is regulated by the family of endogenous tissue inhibitors of metalloproteinases (TIMPs). In addition to the inhibitory role against MMPs, TIMPs may participate in the MMP activation. Moreover, TIMP2 is able to suppress endothelial cell proliferation in response to angiogenic factors, while TIMP3 has the ability to interact with VEGFR2 and block VEGF binding [16,17]. Growth factors such as VEGF, FGF, and PDGF can stimulate production of MMPs [10]. The overexpression of MMPs, observed in many cancers, has been found to correlate with tumor aggressiveness and poor prognosis [18,19]. 

Growing evidence suggests that common germline alterations, such as single nucleotide polymorphisms (SNPs), in angiogenesis-regulating genes may not only increase individual susceptibility to cancer, but may also be implicated in modulating sensitivity to anticancer treatment, thereby affecting therapy results and patient survival [17,20,21,22]. In HNSCC, very few studies have so far addressed the role of SNPs in angiogenesis genes in the context of treatment outcome and prognosis. In a previous report, we demonstrated the predictive and prognostic potential of inherited genetic variants in the ANGPT2/TEK and VEGF/VEGFR2 systems in HNSCC [21]. In the present study, we aimed to evaluate the possible association between a panel of 19 variants in the *FGF2*, *FGFR2*, *PDGFB*, *PDGFRA*, *PDGFRB*, *MMP2*, *MMP9*, *TIMP1*, *TIMP2,* and *TIMP3* genes and the clinical outcomes in non-surgically treated HNSCC patients who received radical RT alone or in combination with cisplatin-based CT.

## 2. Materials and Methods

### 2.1. Patients 

The study group comprised 422 Caucasian patients diagnosed with primary T1–4N0–3M0 HNSCC of the larynx (LSCC), oropharynx (OPSCC) or hypopharynx (HPSCC). The patient characteristics are shown in Table S1. There were 290 (69%) patients with stage III–IVB. Most of the patients were males (80%), cigarette smokers (80%), and alcohol users (77%). All subjects had a WHO performance status of 0 or 1. The treatment and follow-up details have been previously described [21]. Briefly, all patients were treated with curative intent with RT alone (*n* = 219, 52%) or combined with cisplatin-based CT given as induction treatment (docetaxel/cisplatin/5-fluorouracil or cisplatin/5-fluorouracil; *n* = 72, 17%) or administered concurrently (*n* = 131, 31%). Patients who received surgery were excluded from the study. Clinical and demographic data were obtained from medical records and the Silesian Cancer Registry. The study endpoints were overall survival (OS), locoregional recurrence-free survival (LRFS), and metastasis-free survival (MFS). OS was calculated from the date of diagnosis to the date of death from any cause, or the last known date alive. LRFS and MFS were defined as the time from treatment completion to clinically detectable local and/or regional recurrence (for LRFS) or distant metastasis (for MFS), or the last examination without evidence of disease. 

### 2.2. SNP Identification

A total of 19 candidate SNPs were analyzed in this study, including rs5757573, rs2285094 in *PDGFB*, rs2228230, rs1800812 in *PDGFRA*, rs2302273, rs246395 in *PDGFRB*, rs1449683, rs1048201 in *FGF*, rs2981582 in *FGFR2*, rs243865, rs7201 in *MMP2*, rs17576, rs17577 in *MMP9*, rs4898, rs2070584 in *TIMP1*, rs2277698, rs7501477 in *TIMP2*, and rs9862, rs9619311 in *TIMP3* (Table S2). These were SNPs that had a minor allele frequency (MAF) ≥10% in the European Caucasian population [23] and functional significance, and/or were located in coding or regulatory regions, and/or were reported as associated with cancer risk or outcome for other solid cancers [22,24,25,26,27,28,29,30,31,32,33,34,35,36,37,38,39,40,41]. Genomic DNA was isolated from frozen peripheral blood with Genomic Maxi AX kit (A&A Biotechnology, Gdynia, Poland). The SNPs were determined using TaqMan SNP Genotyping Assays (Applied Biosystems, Foster City, CA, USA), according to the manufacturer’s standard protocol. Genotyping was repeated in 50 randomly selected samples, and the concordance was 100%.

### 2.3. Statistical Analysis

The associations between SNPs and survival endpoints were examined using the Kaplan–Meier method and log-rank test. All SNPs were tested under dominant, recessive, and codominant genetic models, and the model with the most significant *p* value was selected for the final analysis. The Cox proportional hazards regression method was used in univariate and multivariate analysis. Multivariate models were adjusted for the following variables: median age at diagnosis (<59 versus ≥59 years), sex (male versus female), cigarette smoking or alcohol use (ever versus never), T stage (T1–2 versus T3–4), N stage (N0 versus N1–3), primary tumor site (LSCC versus OPSCC versus HPSCC), CT use (yes versus no), local and regional relapse (for OS and MFS only; yes versus no), as well as metastasis and second primary cancer, SPC (for OS only; yes versus no). Backward stepwise regression was performed to identify independent risk factors. The proportional hazards assumption was examined using Schoenfeld residuals. Since the assumption was not met in some cases, all hazard ratios should be interpreted as weighted averages of the true values over the follow-up period [42]. A Spearman’s correlation and Pearson’s chi-square test were used to evaluate the associations between variables. To account for multiple testing, the Bonferroni correction was applied, with the significance level set at ≤0.003. However, given the exploratory character of this study, uncorrected *p* values were presented, and *p* ≤ 0.05 was considered statistically significant. All tests were two-sided and Statistica 13.1 (TIBCO Software Inc., Palo Alto, CA, USA) was used for calculations. 

## 3. Results

During the median follow-up period of 72 months, 125 patients (30%) had locoregional recurrence, 48 patients (11%) developed metastasis, and 198 died (47%). The median OS was 105 months in the group treated with RT alone and 70 months in the combination treatment subgroup. The median LRFS and MFS were not reached. Patient baseline characteristics are shown in Table S1. The genotype frequencies were consistent with the Hardy-Weinberg equilibrium (HWE) except for the rs2285094, rs1800812, rs4898 and rs2070584 SNPs, which were excluded from further analysis (Table S2).

**Figure 1:**
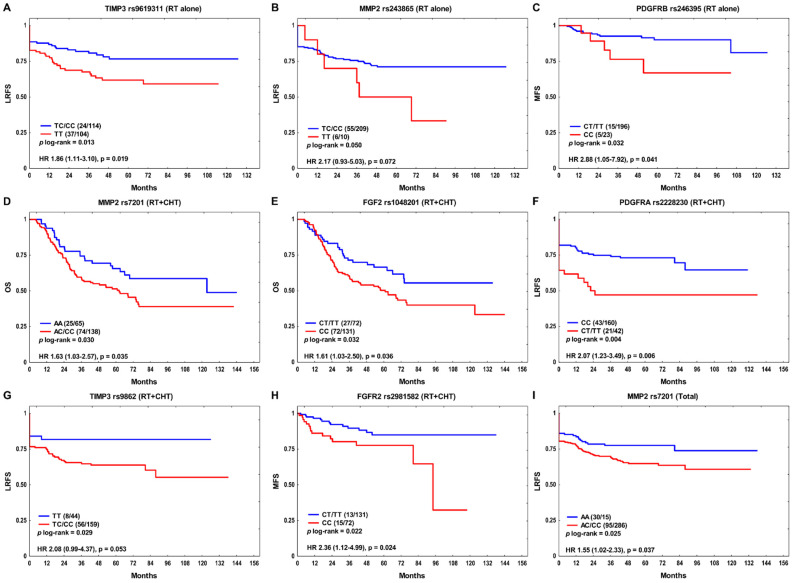
The Kaplan–Meier plots according to SNPs with *p* ≤ 0.05 in univariate analysis for: (**A**,**B**) locoregional recurrence-free survival (LRFS) and (**C**) metastasis-free survival (MFS) in the RT alone subgroup; (**D**,**E**) overall survival (OS); (**F**,**G**) LRFS and (**H**) MFS in the combination treatment subgroup (RT + CHT); and (**I**) LRFS in the whole group. Number of events and *n* are shown in the brackets.

For greater homogeneity in terms of treatment, the data analysis was carried out separately in the subgroup treated with RT alone (*n* = 219) and in patients receiving combined therapy (RT + CT, *n* = 203), as well as in the entire group of patients. In the univariate analysis, *TIMP3* rs9619311, *MMP2* rs243865, and *PDGFRB* rs246395 SNPs were associated with clinical outcome in the RT alone subgroup. Both the rs9619311 TT and rs243865 TT homozygotes showed shorter LRFS than C allele carriers (*p* log-rank = 0.013, hazard ratio (HR) 1.86, *p* = 0.019 and *p* log-rank = 0.050, HR 2.17, *p* = 0.072, respectively; Figure 1A,B). Patients with *PDGFRB* rs246395 CC genotype were at nearly three-fold higher risk of metastasis compared to those with the T allele (*p* log-rank = 0.032, HR 2.88, *p* = 0.041; Figure 1C). In the RT + CT subgroup, *MMP2* rs7201 C and *FGF2* rs1048201 CC were associated with an unfavorable OS (*p* log-rank = 0.030, HR 1.63, *p* = 0.035 and *p* log-rank = 0.032, HR 1.61, *p* = 0.036, respectively; Figure 1D,E). The *PDGFRA* rs2228230 T variant demonstrated a strong association with an increased risk of locoregional relapse (*p* log-rank = 0.004, HR 2.07, *p* = 0.006; Figure 1F). The *TIMP3* rs9862 C carriers showed decreased LRFS (*p* log-rank = 0.029, HR 2.08, *p* = 0.053), while *FGFR2* rs2981582 CC homozygotes were at elevated risk of distant failure (*p* log-rank = 0.022, HR 2.36, *p* = 0.024) (Figure 1G,H). In the whole group, the *MMP2* rs7201 C variant conferred an increased risk of locoregional recurrence (*p* log-rank = 0.025, HR 1.55, *p* = 0.037; Figure 1I), while the association of *TIMP2* rs7501477 T allele with poor LRFS was only marginally significant (*p* log-rank = 0.068, HR 1.40, 95% confidence interval (CI) 0.95–2.07, *p* = 0.085. None of these associations remained statistically significant after the Bonferroni correction.

Subsequently, the effect of six of the above SNPs on the outcome was confirmed in multivariate models integrating genetic, clinical, and demographic factors (Table 1). In the RT alone subset, *MMP2* rs243865 TT homozygotes had an almost three-fold higher risk of locoregional recurrence compared to variant C carriers (HR 2.92, *p* = 0.019), while the *PDGFRB* rs246395 CC genotype was associated with an over three-fold increase in the risk of metastasis (HR 3.06, *p* = 0.041). In the combination treatment subgroup, patients with *FGF2* rs1048201 CC showed a higher risk of death (HR 1.66, *p* = 0.039), while *PDGFRA* rs2228230 T allele carriers were at a significantly increased risk of locoregional relapse (HR 2.49, *p* = 0.001). The *MMP2* rs7201 C and *TIMP2* rs7501477 T alleles were associated with elevated risk of locoregional failure in the entire cohort (HR 1.59, *p* = 0.032 and HR 1.49, *p* = 0.045, respectively). Only the effect of the *PDGFRA* rs2228230 T variant on LRFS survived the correction for multiple testing (Bonferroni adjusted *p* = 0.015). 

The final multivariate models for OS, LRFS, and MFS are presented in Table 2. The analysis identified five SNPs as independent risk factors affecting clinical outcome in the studied HNSCC cohort. In patients treated with RT alone, MMP2 rs243865 TT was a predictor of poor LRFS, together with T3–4, N > 0 and non-oropharyngeal primary site, whereas the *PDGFRB* rs246395 CC genotype and regional recurrence were independent risk factors for shorter MFS. In the RT + CT subgroup, *FGF2* rs1048201 CC, in addition to HPSCC local and regional recurrence, SPC and alcohol use independently predicted unfavorable OS. The *PDGFRA* rs2228230 T variant and non-OPSCC were independent risk factors for poor LRFS in these patients. In all patients, only *MMP2* rs7201 C was found to be an indicator of shorter LRFS, together with T3–4, N > 0 and non-OPSCC. 

**Figure 2:**
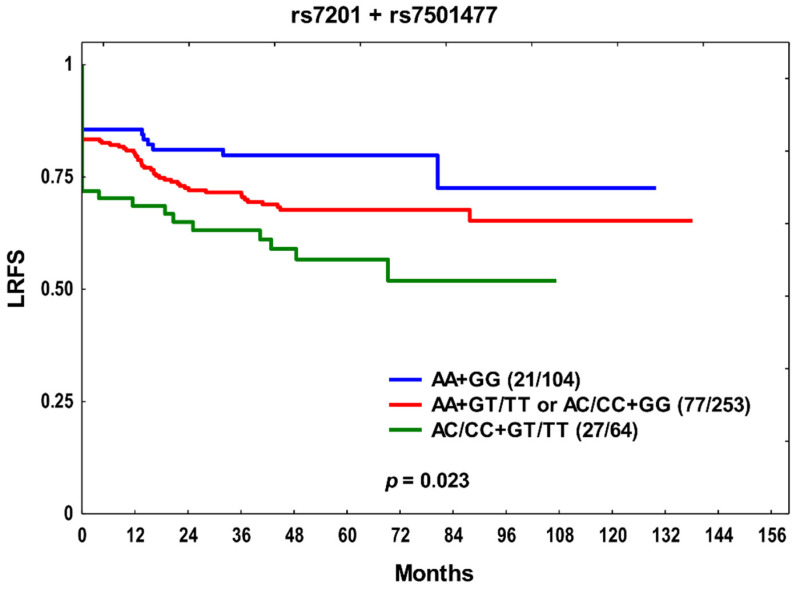
The Kaplan–Meier plot showing combined effects of *MMP2* rs7201 and *TIMP2* rs7501477 on locoregional recurrence-free survival (LRFS) in the studied group. Number of events and *n* are shown in the brackets.

Next, the cumulative effect of unfavorable genotypes on treatment outcomes was assessed. The SNPs with *p* ≤ 0.05 in multivariate analysis were included; therefore, the only combination to be studied comprised rs7201 and rs7501477 in relation to LRFS in the whole group (see Table 1). The rs7201 C and rs7501477 T were assumed to be risk alleles. Patients with both unfavorable variants (i.e., rs7201 AC/CC + rs7501477 GT/TT) had shorter LRFS than carriers of other variant combinations (Figure 2). The rs7201 AC/CC + rs7501477 GT/TT combination was associated with an over two-fold increased genetic risk of locoregional recurrence (HR 2.21, 95% CI 1.25–3.91, *p* = 0.006). The combination was also an independent genetic predictor of unfavorable LRFS (HR 1.67, 95% CI 1.09–2.57, *p* = 0.020), together with clinical features such as T3–4, N > 0, and non-OPSCC. Furthermore, when we tested the rs7201/rs7501477 combination in both treatment subgroups, the AC/CC + GT/TT elevated the risk of locoregional relapse (HR 2.43, 95% CI 1.11–5.29, *p* = 0.026), and it was an independent risk factor for LRFS in the RT + CT subset (HR 1.79, 95% CI 1.00–3.19, *p* = 0.050).

## 4. Discussion

The individual angiogenic potential of the patient may be of great importance for the natural course of the disease and the effectiveness of radiotherapy, as well as systemic, treatment [20]. To date, however, relatively little is known about the impact of common germline variation in genes regulating angiogenesis on therapy outcomes in cancer, especially in HNSCC, and the existing data are often inconsistent. In this study, we identified *FGF2* rs1048201, *PDGFRB* rs246395, *PDGFRA* rs2228230, *MMP2* rs243865, rs7201, and *TIMP2* rs7501477 as predictors of the clinical outcome in HNSCC patients receiving radiotherapy alone or combined with chemotherapy. In multivariate analysis, four of these SNPs were associated with LRFS, one with MFS, and one with OS. Furthermore, the rs1048201 CC, rs2228230 T, rs246395 CC, rs243865 TT, and rs7201 C showed an independent negative effect on the outcome in the final models.

In our HNSCC group, *FGF2* rs1048201 was found to be the only SNP related to OS as observed in the subset treated with combination therapy. The rs1048201 CC genotype increased the risk of death in these patients in a multivariate model. To the best of our knowledge, this SNP has not been studied in cancer before. However, several reports have shown its importance in other pathological conditions such as osteoporosis, non-syndromic orofacial cleft, or diabetic peripheral neuropathy [24,43,44]. The rs1048201 C>T is located in the 3′ untranslated region (UTR) involved in controlling mRNA stability, localization, and translation. The SNP may alter the potential target site for several microRNAs (miRNAs), e.g., hsa-miR-496 [24], hsa-miR-196a-3p [44], and hsa-miR-545 [43], implying its role in the regulation of gene expression. FGF2 aberrant expression has been found in a variety of human malignancies [12]. For example, in head and neck [45,46,47] and lung [48,49] cancers, elevated FGF2 levels in tumor or serum have been correlated with an aggressive phenotype and unfavorable prognosis. Deregulated FGF/FGFR signaling promotes disease progression by increasing angiogenesis and driving the growth, migration, and invasion of cancer cells. Thus, the impact of the rs1048201 SNP on a long-term endpoint such as OS, found in the combination treatment group, could be supported by the direct effect of FGF2 on tumor survival and metastasis. Moreover, it has been shown that upregulated FGF2 confers resistance to anticancer drugs, including cisplatin [50,51,52]. It is therefore plausible that this SNP is of some importance in response to systemic therapy, and in-depth functional studies in the context of cancer treatment would be warranted.

In the present study, the effect on MFS was only noted for *PDGFRB* rs246395 SNP. The CC genotype was an independent prognostic factor associated with an almost three-fold increase in the risk of distant failure after RT alone. Although rs246395 is a synonymous SNP at codon 867 (L867L) in exon 19 and therefore, should not directly affect the amino acid sequence of the protein, it may nevertheless influence mRNA splicing, stability, and structure, as well as protein translation and folding [53]. Similar to our observations, the only study on this SNP demonstrated shorter survival in colorectal cancer patients carrying the C variant [27]. In addition, the CC genotype was correlated with increased PDGFRB protein levels and pathway activation in colorectal cancer cell lines. PDGFRB upregulation has been linked to poor outcome, treatment resistance, and metastasis in several cancers [54,55,56]. Recently, in a large study on early-stage breast cancer, high PDGFRB expression was associated with the risk of recurrence after RT [57]. In oral cancer, a positive correlation was found between elevated PDGFRB levels and lymph node metastases [58]. PDGFRB signaling is implicated in covering new blood vessels with pericytes, providing their remodeling, stabilization, and maturation, as well as the regulation of vascular perfusion, contributing to tumor growth [59,60]. It can be speculated that rs246395 SNP (and/or other variants in linkage disequilibrium, LD) may influence this process by modifying PDGFRB protein function, resulting, for example, in perturbed pericyte–endothelial cell–cell interactions and an increased likelihood of metastatic spread.

Another interesting finding of our study was the strong effect of *PDGFRA* rs2228230 on the risk of locoregional recurrence after combination treatment. Importantly, this effect remained significant even after adjusting for multiple comparisons using the conservative Bonferroni method. The magnitude of risk was also larger than that of the independent clinical risk factor in the model. The rs2228230 V824V is located in exon 18, encoding the tyrosine kinase domain II. It is the second synonymous SNP relevant for predicting treatment outcomes in our HNSCC cohort, although data on its functional significance and prognostic role in cancer are very limited and contradictory. Consistent with our findings, in a Spanish study, the rs2228230 TT genotype was correlated with unfavorable disease-free survival rates in patients with renal cell carcinoma [26]. In contrast, a Chinese report showed a protective effect of variant T on OS and progression-free survival in acral melanoma [25]. In the same study, the T allele was associated with decreased stability and expression of *PDGFRA* mRNA and protein, as well as reduced downstream signaling activity. Nevertheless, PDGFRA overexpression was observed in many cancers, which correlated with malignant progression [11,61,62]. For example, high levels of PDGFRA have been associated with regional metastasis and decreased survival in oral carcinoma [58,63]. Therefore, it seems that the sparse data obtained so far on rs2228230 suggest that the effect of this synonymous SNP is likely context-dependent and may vary according to e.g., the ethnic origin of the population and/or type of cancer.

Furthermore, we demonstrated that the *MMP2* gene variants were independently associated with locoregional failure in our HNSCC group. The *MMP2* SNPs have been studied fairly extensively in the context of susceptibility to various cancers and non-malignant pathologies, but the results were inconclusive [64,65]. In contrast, there is little data on these SNPs as risk factors for cancer progression and clinical outcome, especially in HNSCC. The rs243865 SNP causes -1306C>T transition in the gene promoter region that abolishes the Sp1 binding site, reducing its transcriptional activity [28]. In the current study, the TT genotype conferred a three-fold increase in the risk of recurrence after RT alone. This corresponds to our previous report on inoperable NSCLC patients receiving RT with or without chemotherapy, in which we found a significant association between the rs243865 T and earlier progression [22]. The T allele also correlated with an unfavorable prognosis in colorectal [66], ER negative breast [67], and cervical cancer [68], while in bladder cancer, the rs243865 T carriers were at an increased risk of recurrence [69]. However, oral cancer patients with T variant showed lower metastasis rates after surgery [70]. In addition, it was shown that the *MMP2* expression levels in HNSCC cell lines and tumors with the CC genotype were higher compared to those carrying the CT genotype [71]. At the same time, variant T was found to be protective with respect to head and neck cancer susceptibility [70,71,72]. Thus, the present study indicates, for the first time, that the rs243865 TT may be a risk factor for HNSCC recurrence. Our findings support the functional importance of this SNP; however, the direction of its effect in terms of different types of cancer and treatments remains to be elucidated. Moreover, the complexity of the MMP2 role in cancer should be mentioned here, since MMP2 may be involved in blocking angiogenesis by cleaving plasminogen and producing angiostatin [15,73].

Finally, we identified *MMP2* rs7201 C and *TIMP2* rs7501477 T variants as predictors of locoregional recurrence in the whole group, both individually and in combination. The rs7201/rs7501477 combination also showed an independent effect on the risk of recurrence in all patients and in the combination treatment subset. The rs7201 is 3′UTR SNP in the miRNA binding site, suggesting its regulatory effect on gene expression, while rs7501477 -4804G>T in the gene promoter region is predicted to create binding sites for several transcription factors that may act as activators or repressors of target genes [74]. It has been found that the rs7201 C variant reduced the silencing effect of miRNA-520 g and was associated with an increased expression level in the reporter assay [29]. Unfortunately, there is no data on the predictive or prognostic value of this SNP in cancer. The only available studies concerned the risk of laryngeal and nasopharyngeal carcinomas and showed no association [75,76]. In turn, the rs7501477 TT was identified as a risk factor for breast cancer in a single study, but with no effect on survival [30]. Both MMP2 and TIMP2 are known to interact with each other in modulating the angiogenic response. By regulating the MMP2 catalytic activity, TIMP2 not only inactivates the active form of the enzyme, but it is also required for the pro-MMP2 activation [77]. It can therefore be assumed that rs7201 and rs7501477 SNPs, by leading to the MMP/TIMP imbalance, as well as possibly affecting MMP2 and TIMP2 functions, may partially contribute to locoregional relapse in HNSCC patients.

In summary, our data demonstrate that common germline alterations in some angiogenesis-related genes may constitute determinants of treatment efficacy and tumor aggressiveness in HNSCC relevant to standard therapy, such as curative RT alone or combined with cisplatin-based CT. The observed effects may be specific to a particular modality of standard treatment. Our findings may also be of some importance in anti-angiogenic therapy and immunotherapy. Moreover, recent data show beneficial effects of combining immune checkpoint inhibitors with anti-angiogenic agents in several cancers [78]. Given the complex interplay between the vasculature and immune systems, and the immunosuppressive role of VEGF and other angiogenic molecules (e.g., FGF2) in the tumor microenvironment [79], it cannot be excluded that variation in angiogenesis genes may contribute to the modulation of these processes, affecting the response to the above-mentioned therapies. As the *FGF2* rs1048201, *PDGFRB* rs246395, *PDGFRA* rs2228230, *MMP2* rs243865, rs7201, and *TIMP2* rs7501477 have not been previously examined in HNSCC in terms of survival and treatment outcome, this is most likely the first report describing their prognostic and predictive value in this type of cancer. Nevertheless, our study has limitations, including a moderate size of the patient group and currently little understanding of the biological mechanisms explaining the observed associations. In addition, it cannot be ruled out that the SNPs we identified are not true causal variants. Hence, their potential clinical relevance should be investigated in large datasets, and functional studies are also required.

## 5. Conclusions

In conclusion, the present study shows that the *FGF2* rs1048201, *PDGFRB* rs246395, *PDGFRA* rs2228230, *MMP2* rs243865, and rs7201 variants may independently predict therapy failure and poor survival in non-surgically treated HNSCC patients receiving radical RT alone or combined with cisplatin-based CT. Information on individual host genetic risk factors could be a valuable complement to the classical clinical factors used to assess the risk of locoregional and distant relapse in HNSCC, ultimately contributing to improved prognosis.

## Figures and Tables

**Table 1:** Multivariate analysis for association of SNPs (only SNPs with *p* ≤ 0.100 are shown) with OS, LRFS, and MFS.

SNP	Genotype	RT Alone		RT + CT		Total	
		HR (95% CI)	*p*	HR (95% CI)	*p*	HR (95% CI)	*p*
**OS**							
rs1048201	CC	0.76 (0.49–1.17)	0.208	1.66 (1.03–2.68)	**0.039**	1.10 (0.81–1.48)	0.544
**LRFS**							
rs2228230	CT/TT	0.83 (0.44–1.57)	0.572	2.49 (1.42–4.36)	**0.001**	1.41 (0.93–2.14)	0.106
rs243865	TT	2.92 (1.20–7.11)	**0.019**	0.74 (0.23–2.42)	0.620	1.38 (0.69–2.73)	0.360
rs7201	AC/CC	1.50 (0.82–2.75)	0.191	1.54 (0.84–2.81)	0.159	1.59 (1.04–2.42)	**0.032**
rs7501477	GT/TT	1.41 (0.79–2.52)	0.250	1.57 (0.91–2.72)	0.107	1.49 (1.01–2.21)	**0.045**
rs9862	TC/CC	0.91 (0.51–1.63)	0.749	2.12 (0.98–4.57)	0.055	1.18 (0.76–1.82)	0.459
**MFS**							
rs246395	CC	3.06 (1.05–8.95)	0.041	0.36 (0.08–1.70)	0.198	1.29 (0.56–2.99)	0.548
rs1048201	CC	3.08 (0.92–10.25)	0.067	1.31 (0.57–3.01)	0.519	1.70 (0.89–3.24)	0.111

RT, radiotherapy; RT + CT, combination treatment; HR, hazard ratio; CI, confidence interval; OS, overall survival; LRFS, locoregional recurrence-free survival; MFS, metastasis free survival; *p* ≤ 0.050 shown in bold.

**Table 2:** Independent risk factors for OS, LRFS, and MFS—the final models.

	RT Alone			RT + CT			Total		
	Variables	HR (95% CI)	*p*	Variables	HR (95% CI)	*p*	Variables	HR (95% CI)	*p*
OS	Stage N > 0 SPC Metastasis Local recurrence	2.24 (1.49–3.36) 2.29 (1.37–3.84) 1.89 (1.08–3.32) 3.95 (2.62–5.97)	0.0001 0.0016 0.026 <1 × 10^−6^	rs1048201 CC Alcohol: ever HPSCC Local recurrence Regional recurrence SPC	1.61 (1.01–2.55) 2.11 (1.18–3.74) 2.01 (1.25–3.24) 5.51 (3.33–9.11) 1.73 (1.06–2.84) 2.07 (1.09–3.93)	0.044 0.011 0.004 <1 × 10^−6^ 0.029 0.026	Alcohol: ever Stage N > 0 Local recurrence Regional recurrence Metastasis SPC	1.51 (1.06–2.16) 1.81 (1.31–2.49) 4.84 (3.52–6.67) 1.49 (1.01–2.18) 1.72 (1.17–2.54) 2.32 (1.56–3.46)	0.024 0.0003 <1 × 10^−6^ 0.044 0.006 4 × 10^−5^
LRFS	rs243865 TT Stage T3–4 Stage N > 0 Non-OPSCC	2.92 (1.23–6.94) 2.97 (1.72–5.14) 2.19 (1.23–3.91) 2.29 (1.20–4.39)	0.015 0.0001 0.008 0.012	rs2228230 CT/TT Non-OPSCC	2.26 (1.33–3.84) 1.74 (1.05–2.87)	0.003 0.032	rs7201 AC/CC Stage T3–4 Stage N > 0 Non-OPSCC	1.56 (1.02–2.37) 1.69 (1.14–2.50) 1.68 (1.11–2.55) 1.66 (1.11–2.49)	0.038 0.008 0.015 0.013
MFS	rs246395 CC Regional recurrence	2.79 (1.01–7.69) 5.56 (1.71–18.13)	0.048 0.004	Regional recurrence	3.65 (1.62–8.22)	0.002	HPSCC Regional recurrence	2.36 (1.13–4.89) 4.60 (2.37–8.92)	0.021 6 × 10^−6^

RT, radiotherapy; RT + CT, combination treatment; HR, hazard ratio; CI, confidence interval; OS, overall survival; LRFS, locoregional recurrence-free survival; MFS, metastasis free survival; HPSCC, hypopharyngeal squamous cell carcinoma; Non-OPSCC, non-oropharyngeal squamous cell carcinoma; SPC, second primary cancer.

## Data Availability

The data are available from the corresponding author on reasonable request.

## Supplementary Materials

The following supporting information can be downloaded at: https://www.mdpi.com/article/10.3390/cancers14071844/s1, Table S1: General characteristics of the studied patients; Table S2: SNPs examined in the study and the genotype distribution in all patients.
