# Supplementary material for: Germline Variants in Angiogenesis-Related Genes Contribute to Clinical Outcome in Head and Neck Squamous Cell Carcinoma
Source: Cancers (Basel). 2022 Apr 6;14(7):1844. doi: 10.3390/cancers14071844 (PMC8997703; doi:10.3390/cancers14071844)
Supplement: Supplementary file 1 [file cancers-14-01844-s001.zip › cancers-1593018-supplementary.pdf]

Table S1. General characteristics of the studied patients.

Parameter	RT alone<br>n = 219	RT + CT<br>n = 203	Total<br>n = 422
Median age at diagnosis			
< 59 years	76 (35%)	120 (59%)	196 (46%)
≥ 59 years	143 (65%)	83 (41%)	226 (54%)
Male	175 (80%)	164 (81%)	339 (80%)
Female	44 (20%)	39 (19%)	83 (20%)
Tumor site			
LSCC	147 (67%)	59 (29%)	206 (49%)
OPSCC	57 (26%)	106 (52%)	163 (39%)
HPSCC	15 (7%)	38 (19%)	53 (12%)
T stage			
T1	49 (22%)	6 (3%)	55 (13%)
T2	106 (49%)	53 (26%)	159 (38%)
T3	48 (22%)	83 (41%)	131 (31%)
T4	16 (7%)	61 (30%)	77 (18%)
N stage			
N0	154 (70%)	33 (16%)	187 (44%)
N1–3	65 (30%)	170 (84%)	235 (56%)
Smoking status			
Never	39 (18%)	46 (23%)	85 (20%)
Ever	180 (82%)	157 (77%)	337 (80%)
Alcohol use [a]			
Never	50 (23%)	47 (23%)	97 (23%)
Ever	168 (77%)	154 (77%)	322 (77%)
Overall survival			
Median (range) months	104 (4–136)	70 (5–144)	74 (4–144)

RT, radiotherapy; RT+CT, combination treatment; LSCC, laryngeal squamous cell carcinoma; OPSCC, oropharyngeal squamous cell carcinoma; HPSCC, hypopharyngeal squamous cell carcinoma; [a] The missing data for three patients – one in the RT subset and two in the RT + CT subset.

Table S2. SNPs examined in the study and the genotype distribution in all patients.

Gene	SNP	Allele	Location	Genotype distribution [a]	Function class [b]	RegDB rank [c]	EUR frequency [d]	MAF	HWE *p*-Value
PDGFRA	rs2228230	Val824Val, 2547C>T	exon 18	318/94/9	S, splicing (ESE/ESS)	5	0.15	0.13	0.51
	rs1800812	–635G>T	promoter	281/118/22	TFBS	2b	0.21	0.19	0.04
PDGFRB	rs2302273	–302G>A	5′UTR	290/110/18	TFBS	1f	0.19	0.17	0.07
	rs246395	Leu867Leu, 2601T>C	exon 19	202/177/43	S, splicing (ESE/ESS)	5	0.30	0.31	0.65
PDGFB	rs5757573	c.64–1743T>C	intron 2	177/188/55	-	1f	0.38	0.35	0.65
	rs2285094	c.602–708T>C	intron 6	72/287/62	-	4	0.46	0.49	$< 1 \times 10^{-6}$
FGF2	rs1449683	Ser52Ser, 156C>T	exon 1	347/70/2	S	2b	0.10	0.09	0.44
	rs1048201	626C>T	3′UTR	268/140/13	miRNA binding site	7	0.18	0.20	0.30
FGFR2	rs2981582	IVS2 + 906C>T	intron 2	156/196/70	-	2b	0.42	0.40	0.53
MMP2	rs243865	–1306C>T	promoter	249/151/22	TFBS	1f	0.27	0.23	0.89
	rs7201	260A>C	3′UTR	135/208/78	TFBS, miRNA binding site	5	0.48	0.43	0.89
MMP9	rs17576	Gln279Arg, 836A>G	exon 6	152/216/54	NS, splicing (ESE/ESS)	4	0.38	0.38	0.09
	rs17577	Arg668Gln, 2003G>A	exon 12	294/118/10	NS, splicing (ESE/ESS), miRNA binding site	2b	0.17	0.16	0.65
TIMP1	rs4898	Phe124Phe, 327T>C	exon 5	193/47/182	S, splicing (ESE/ESS), miRNA binding site	4	0.46	0.49	$< 1 \times 10^{-6}$
	rs2070584	g.9830T>G	3′UTR	201/47/174	-	2b	0.45	0.47	$< 1 \times 10^{-6}$
TIMP2	rs2277698	Ser101Ser, 303C>T	exon 3	328/87/7	S, splicing (ESE/ESS)	5	0.13	0.12	0.66
	rs7501477	–4804G>T	promoter	327/87/8	TFBS	2b	0.10	0.12	0.44
TIMP3	rs9862	His83His, 249C>T	exon 3	114/208/100	S, splicing (ESE/ESS)	3a	0.49	0.48	0.79
	rs9619311	–1296T>C	promoter	195/179/46	TFBS	4	0.31	0.32	0.61

SNP, single nucleotide polymorphism; MAF, minor allele frequency in the studied group (n = 422); HWE, Hardy-Weinberg equilibrium; S, synonymous; NS, nonsynonymous; TFBS, transcription factor binding site; ESE/ESS; exonic splicing enhancer/exonic splicing silencer; [a] The genotype distribution shown in order of common homozygote/heterozygote/variant homozygote; [b] According to https://snpinfo.niehs.nih.gov/snpinfo/snpfunc.html; [c] According to www.regulomedb.org; [d] SNP frequency in European population according to www.ensembl.org.
